# Eye Salvage and Vision Preservation in High‐Risk Intraocular Retinoblastoma Patients: Long‐Term Results From the Prospective Phase II AIEOP RTB 012 Study

**DOI:** 10.1002/cam4.71188

**Published:** 2025-09-02

**Authors:** Ida Russo, Valentina Di Ruscio, Maria Antonietta De Ioris, Giada Del Baldo, Maria Debora De Pasquale, Paola Valente, Enrico Opocher, Raffaele Parrozzani, Angela Di Giannatale, Maria Giuseppina Cefalo, Annalisa Serra, Giuseppe Maria Milano, Daniela Longo, Alessia Carboni, Rita De Vito, Gianluigi Natali, Gemma D'Elia, Gianni Bisogno, Angela Mastronuzzi, Raffaele Cozza, Rita Alaggio, Antonino Romanzo, Luca Buzzonetti, Franco Locatelli

**Affiliations:** ^1^ Onco‐Hematology, Cell Therapy, Gene Therapies and Hemopoietic Transplant Bambino Gesù Children's Hospital, IRCCS Rome Italy; ^2^ Ophthalmology Unit Bambino Gesù IRCCS Children's Hospital Passoscuro, Rome Italy; ^3^ Hematology Oncology Division, Department of Women's and Children's Health University of Padua Padua Italy; ^4^ Department of Ophthalmology University of Padua Padua Italy; ^5^ Neuroradiology Unit “Bambino Gesù” Children's Hospital IRCCS Rome Italy; ^6^ Pathology Unit, Department of Laboratories Bambino Gesu Children's Hospital, IRCCS Rome Italy; ^7^ Interventional Radiology Unit, Department of Surgery Bambino Gesù Children's Hospital Rome Italy; ^8^ Laboratory of Medical Genetics, Molecular Genetics Unit Bambino Gesù Children Hospital, IRCCS Rome Italy; ^9^ Department of Life Sciences and Public Health Catholic University of the Sacred Heart Rome Italy

**Keywords:** high‐risk disease, intraocular, ocular survival, retinoblastoma, visual outcome

## Abstract

**Introduction:**

This study presents the outcomes of high‐risk group retinoblastoma (Rb) patients enrolled in the AIEOP RTB 012 Protocol.

**Methods:**

Patients with intraocular unilateral Rb classified as group C/D according to “International Intraocular Retinoblastoma Classification” (IIRC), as well as those with bilateral Rb with at least one eye group C/D/E‐IIRC, were treated with four cycles of carboplatin/etoposide combined with focal treatments.

The primary endpoint was to evaluate ocular event‐free survival (EFS) and overall survival (OS), where events were defined as the need for radiotherapy, eye enucleation, and second‐line treatment. Visual acuity (VA) was assessed in all available cases.

**Results:**

Between February 2012 and September 2017, 60 patients were enrolled (88 eyes), 32 unilateral Rb, 28 bilateral. Twelve eyes were classified as group A/B, 15 group C, 40 group D, and 21 group E. At a median follow‐up of 8.71 years, 42/88 eyes were preserved. The 2‐ and 5‐year eye EFS rates were 31.1% (95% CI: 24.4–44.0) and 29.5% (95% CI: 20.4–39.2), respectively. The corresponding 2‐ and 5‐year eye OS rates were 63.3% (95% CI: 52.7–72.7) and 48.9% (95% CI: 38.1–58.8). Ocular survival significantly differed across IIRC groups, with 2‐ and 5‐year survival rates of 100% for group A/B, 86.7% and 73.3% for group C, 65% and 42.5% for group D, and 23.8% and 14.3% for group E, respectively (*p* < 0.05). No severe toxicity was reported. Among 28 bilateral Rb patients, 12 had VA of at least 20/30 according to the Snellen chart.

**Conclusion:**

The AIEOP RTB 012 protocol has proven to be both safe and effective, ensuring a favorable final VA.

## 1 Introduction

Retinoblastoma (Rb) is a rare, potentially life‐threatening childhood cancer with a stable incidence worldwide, estimated to be approximately 1:16,000 to 18,000 live births [1].

In the past three decades, conservative approaches for intraocular disease have evolved significantly. Focal ocular treatments, including cryotherapy, thermotherapy, and plaque radiotherapy, are now commonly integrated with chemotherapy to guarantee ocular survival and preserve visual function. This strategy aims to minimize the need for aggressive treatments such as external beam radiotherapy (EBRT), which is particularly undesirable in children with germline Rb1 alteration due to the increased risk of secondary malignancies. During the period of our study protocol, ocular‐directed chemotherapy techniques, such as intra‐arterial (IAC) and intravitreal (IVTC), were still in their early stages and often used as second‐line treatments [2, 3]. The most widely used systemic chemotherapy scheme consists of carboplatin and etoposide [4, 5, 6]. We report the results of the Italian multicenter prospective phase II study‐AIEOP RTB 012, focusing on long‐term ocular preservation rate and visual acuity (VA).

## 2 Materials and Methods

### 2.1 Patient Population

The AIEOP RTB 012 protocol, designed for treatment of patients with intraocular Rb, was approved by the Bambino Gesù Children Hospital Ethics Committee and was open for enrollment from January 1, 2012 to December 31, 2017 (EudraCT Number: 2011‐006109‐85). Written informed consent was obtained from each participant's parent or legal guardian. Eyes were categorized according to the intraocular disease burden in accordance with the International Intraocular Retinoblastoma Classification (IIRC) [7] and divided into two groups: high‐risk—unilateral Rb classified as group C/D‐IIRC and/or bilateral Rb with at least one eye group C, D, or E‐IIRC; low‐risk—unilateral Rb classified as group A/B‐IIRC and/or bilateral Rb with both eyes group A/B‐IIRC. Patients with unilateral intraocular disease group E‐IIRC were excluded from the trial, since they underwent primary enucleation. Patients with extraocular disease were also excluded. The enrollment target was not met by the low‐risk cohort, and as a result, it was not analyzed. Additional eligibility criteria included life expectancy longer than 8 weeks, an Eastern Cooperative Oncology Group (ECOG) performance status of 0 to 2, and adequate liver and renal function defined as total bilirubin, Aspartate Aminotransferase‐AST, Alanine Aminotransferase‐ALT, and serum creatinine levels below three times the upper normal limit. Genetic counseling and testing for germline Rb1 alteration was provided for all patients.

### 2.2 Treatment Plan

Protocol treatment consisted of four cycles of carboplatin and etoposide, administered at 21‐day intervals, either alone or in combination with focal treatments starting from the third cycle. Focal therapies included cryotherapy, thermotherapy, and brachytherapy. In cases of persistent disease, two additional courses of carboplatin were administered in association with thermotherapy (ThermoChemoTherapy—TCTh modality) (Table 1).

**TABLE 1:** Treatment plan.

	Treatments	Chemotherapy doses
High risk group‐IIRC	Carbo/VP16 × 4 +/− Carbo (TCTh) × 2 +/− FT	Carboplatin: Age > 12 months, weight > 10 kg: 560 mg/m^2^ Day 1 Age < 12 months and weight < 10 kg: 18.6 mg/kg Day 1 Age < 1 month and weight < 5 kg: reduction of 25% of expected dose pro/kg, with the possibility of increase if no toxicities are reported VP16: Age > 12 months and weight > 10 kg: 150 mg/m^2^ Day 1 and 2 Age < 12 months and weight < 10 kg: 5 mg/kg Day 1 and 2 Age < 1 month and weight < 5 kg: reduction of 25% of expected dose pro/kg, with the possibility of increase if no toxicities are reported

Abbreviations: Carbo, carboplatin; FT, Focal therapies including thermotherapy, cryotherapy, brachytherapy; IIRC, International Intraocular Retinoblastoma Classification; TCTh, ThermoChemoTherapy; VP16, etoposide.

### 2.3 Ophthalmological Evaluation

Patient follow‐up included funduscopy (under general anesthesia up to the age of 4), monthly during the first year after the last event, and then every 2 to 3 months. Fundus photographs, regularly performed with the RetCam, were analyzed with respect to choroidal ischemia at the macular and extramacular region, retinal pigment changes, and retinopathy. VA assessments were carried out for all patients from the age of 4 to 5 years, with a standardized Snellen ‘E’ chart (4–5 years) and Snellen chart (> 6 years).

### 2.4 Dose Modifications and Adverse Events

Adverse events (grade 3 to 5, or any unexpected grade 2) were monitored according to the National Cancer Institute Common Terminology Criteria for Adverse Events (version 4.0) [8]. Hematologic parameters were assessed before each chemotherapy course, requiring an absolute neutrophil count greater than 750/mm^3^ and platelet count above 75,000/mm^3^, without the need for transfusion support. If chemotherapy was delayed by more than 7 days in two consecutive courses due to neutropenia or thrombocytopenia, the doses of all cytotoxic agents were reduced by 25% in subsequent courses. Renal function was evaluated by determining the glomerular filtration rate (GFR) at baseline and after the third dose of carboplatin using the Schwartz formula [9]. Auditory function was assessed before treatment initiation, during therapy, and annually during follow‐up following the Brock grading system [10].

### 2.5 Statistical Design and Analysis

The AIEOP RTB 012 protocol was a single‐arm, two‐stage phase II study designed to evaluate response to systemic chemotherapy combined with focal ocular treatments as its primary objective. Data entry and cleaning were conducted using Microsoft Excel, while statistical analyses were performed using GraphPad Prism software (version 10.0). Statistical analysis was based on individual eyes rather than patients as the sample unit. Population Overall Survival (OS) was defined as the time from diagnosis to death or last follow‐up. Ocular OS was defined per eye as the time from study entry to enucleation or last follow‐up. Ocular Event Free Survival (EFS) was defined per eye as the time from study entry to the occurrence of the first event or last follow‐up for eyes without events. Events were defined as enucleation, EBRT, or any other treatment not included in the original protocol design. All eligible and evaluable patients were included in EFS and OS analyses. Global and ocular OS and EFS were estimated using the Kaplan–Meier method with 95% confidence intervals. Comparison of survival distribution between the intraocular classification groups was performed using the *log‐rank test*. The significance threshold (*α*‐risk) was set at 5%.

## 3 Results

### 3.1 Patients

Between February 2012 and September 2017, 60 patients were enrolled in the high‐risk group of AIEOP RTB 012 Protocol. The median age at diagnosis was 39 months (range: 0–47 months), with a male‐to‐female ratio of 1.22. At onset, 28 patients presented with bilateral disease, while 32 had unilateral Rb, resulting in a total of 88 eyes treated and available for protocol evaluation. Among 32 patients with unilateral Rb, 7 eyes were group C‐IIRC, and 25 eyes were group D‐IIRC; among 28 patients with bilateral disease, the most frequent groups were E and D‐IIRC (21 and 15 eyes affected, respectively), 8 eyes were group C‐IIRC, 8 eyes were group A‐IIRC, and 4 eyes were group B‐IIRC. Patient characteristics are provided in Table 2. Genetic analysis identified a Rb1 germline alteration in 29 out of 60 patients (48%). Among them, 15 cases were detected by next‐generation sequencing, while 14 cases were identified using multiplex ligation‐dependent probe amplification.

**TABLE 2:** Eyes' characteristics and treatments.

		Right eye				Left eye				
Pt	Initial CT (n° of cycles)	IIRC group	Focal therapy (Y/N, specify)	Relapse/Salvage therapy	Eye‐O at last f‐up	IIRC group	Focal therapy (Y/N, specify)	Relapse/Salvage therapy	Eye‐O at last F‐up	Pt‐O at last F‐up
1	2‐CT (4)	C	Y, TTh, Cryoth, TCTh	N	Saved	A	Y, TTh	N	Saved	A
2	2‐CT (4)	A	Y, TCTh, TTh	N	Saved	E	N	N	Enucl	A
3	2‐CT (4)	E	Y, Cryoth, TCTh, TTh	Y, 3‐IVT, 3‐IAC (*1)	Enucl	C	Y, Cryoth, TCTh, TTh	Y, 15 IVT, 6 IAC (*1)	Enucl	A
4	2‐CT (4)	No RB	—	—	—	D	Y, Cryoth, TCTh, TTh	N	Enucl	A
5	2‐CT (4)	D	Y, TTh, TCTh, CryoTh, BrachyTh	Y, 8 IVT, 3 IAC (*1)	Enucl	No RB	—	—	—	A
6	2‐CT (4)	D	Y, TTh, TCTh, CryoTh, BrachyTh	Y, 9 IVT, 3 IAC (*1)	Enucl	No RB	—	—	—	A
7	2‐CT (4)	A	Y, TTh	N	Saved	C	Y, TTh	Y, 3 IAC (1*)	Saved	A
8	2‐CT (4)	D	Y, TTh	N	Saved	No RB	—	—	—	A
9	2‐CT (4)	D	Y, TTh, TCTh, CryoTh	Y, 7 IVT	Saved	E	N (enucl)	N	Enucl	A
10	2‐CT (4)	No RB	—	—	—	D	Y, TTh, TCTh, CryoTh, BrachyTh	N	Saved	A
11	2‐CT (4)	C	Y, TCTh, TTh, CryoTh, BrachyTh	N	Enucl	No RB	—	—	—	A
12	2‐CT (4)	D	Y, TCTh, CryoTh, BrachyTh	Y, 3 IAC (1*)	Enucl	No RB	—	—	—	A
13	2‐CT (4)	E	Y, TCTh, TTh, CryoTh	Y, 6 IAC (1*)	Enucl	B	Y, TCTh, TTh, CryoTh, BrachyTh	N	Saved	A
14	2‐CT (4)	No RB	—	—	—	C	Y, TCTh, TTh, CryoTh, BrachyTh	N	Saved	A
15	2‐CT (4)	No RB	—	—	—	D	Y, TCTh, TTh	N	Saved	A
16	2‐CT (4)	No RB	—	—	—	D	Y, TCTh, TTh, CryoTh	Y, 4 IVT, 7 IAC (2*)	Enucl	D
17	2‐CT (4)	D	Y, TCTh, TTh, CryoTh	Y, 3 IAC (1*)	Enucl	No RB	—	—	—	A
18	2‐CT (4)	D	Y, TCTh, TTh, CryoTh	Y, 7 IVT	Enucl	No RB	—	—	—	A
19	2‐CT (4)	D	Y, TCTh, TTh, CryoTh, BrachyTh	Y, 3 IAC (1*)	Enucl	No RB	—	—	—	A
20	2‐CT (4)	No RB	—	—	—	4	Y, TCTh, TTh	Y, 4 IVT, 3 IAC (1*)	Enucl	A
21	2‐CT (4)	C	Y, TTh, TCTh	Y, 3 IVT	Saved	E	N	N	Enucl	A
22	2‐CT (4)	No RB	—	—	—	D	Y, TTh, CryoTh	Y, 3 IAC (1*), 3 IAC (2*), 3 IVT	Enucl	D
23	2‐CT (4)	D	Y, TTh, CryoTh	Y, 3 IVT, 3 IAC (1*), 3 IAC (2*)	Enucl	No RB	—	—	—	A
24	2‐CT (4)	E	Y, TCTh, TTh, CryoTh	Y, 3 IVT	Enucl	E	Y, TCTh, TTh, CryoTh	Y, 4 IVT, 3 IAC (1*)	Enucl	A
25	2‐CT (4)	A	Y, TTh, CryoTh	N	Saved	D	Y, TCTh, TTh, CryoTh, BrachyTh	N	Saved	A
26	2‐CT (4)	C	Y, TCTh, TTh, CryoTh, BrachyTh	Y, 3 IVT	Saved	B	Y, TCTh, TTh, CryoTh, BrachyTh	N	Saved	A
27	2‐CT (4)	E	Y, TCTh, TTh	Y, 4 IVT, 3 IAC (1*)	Enucl	D	Y, TCTh, TTh, CryoTh, BrachyTh	Y, 3 IAC (1*)	Saved	A
28	2‐CT (4)	C	Y, TTh	N	Saved	No RB	—	—	—	A
29	2‐CT (4)	C	Y, TCTh	N	Saved	No RB	—	—	—	A
30	2‐CT (4)	E	Y, TTh, CryoTh	Y, 3 IVT, 3 IAC (1*)	Enucl	D	Y, TTh, CryoTh, BrachyTh	Y, 3 IAC (1*)	Saved	A
31	2‐CT (4)	C	Y, TTh, CryoTh	Y, 6 IVT, 3 IAC (1*), 6 IAC (2*)	Enucl	No RB	—	—	—	A
32	2‐CT (4)	E	N	N	Enucl	E	Y, TTh, CryoTh	Y, 6 IVT, 3 IAC (1*), 1 IAC (2*)	Enucl	A
33	2‐CT (4)	C	Y, TTh, CryoTh	Y, 2 IVT	Saved	E	Y, TTh, CryoTh	N	Enucl	A
34	2‐CT (4)	E	N	N	Enucl	C	Y, TCTh, TTh, CryoTh, BrachyTh	N	Saved	A
35	2‐CT (4)	D	Y, TCTh, TTh, CryoTh, BrachyTh	Y, 2 IVT, 3 IAC (1*), 3 IAC (2*)	Enucl	E	N	N	Enucl	A
36	2‐CT (4)	D	Y, TCTh, CryoTh	N	Saved	E	N	N	Enucl	A
37	2‐CT (4)	No RB	—	—	—	D	Y, TTh, BrachyTh, CryoTh	Y, 6 IVT	Enucl	A
38	2‐CT (4)	No RB	—	—	—	D	Y, TTh, TCTh	Y, 13 IVT	Saved	A
39	2‐CT (4)	D	Y, TTh	Y, 6 IVT, 6 IAC (1*)	Enucl	No RB	—	—	—	A
40	2‐CT (4)	E	Y, TCTh, TTh, CryoTh, BrachyTh	Y, 3 IAC (1*)	Saved	B	Y, TCTh, TTh	N	Saved	A
41	2‐CT (4)	D	Y, TCTh, TTh	N	Saved	A	Y, TTh, CryoTh	N	Saved	A
42	2‐CT (4)	C	Y, TCTh, TTh, CryoTh, BrachyTh	Y, 3 IAC (1*), 2 IVT	Saved	No RB	—	—	—	A
43	2‐CT (4)	E	N	N	Enucl	D	Y, TTh	Y, 3 IVT	Saved	A
44	2‐CT (4)	D	Y, BrachyTh	N	Saved	No RB	—	—	—	A
45	2‐CT (4)	No RB	—	—	—	D	Y, TTh, CryoTh	N	Saved	A
46	2‐CT (4)	No RB	—	—	—	D	Y, TTh, BrachyTh	Y	Enucl	A
47	2‐CT (4)	A	N	N	Saved	D	Y, TTh, BrachyTh	Y	Enucl	A
48	2‐CT (4)	D	Y, CryoTh, TTh	N	Enucl	No RB	—	—	—	A
49	2‐CT (4)	No RB	—	—	—	D	N	N	Saved	A
50	2‐CT (4)	C	Y, TTh, CryoTh, BrachyTh	N	Saved	A	Y, TTh, CryoTh	N	Saved	A
51	2‐CT (4)	No RB	—	—	—	D	Y, BrachyTh	Y	Enucl	A
52	2‐CT (4)	No RB	—	—	—	D	Y, BrachyTh	Y, 8 IVT	Enucl	A
53	2‐CT (4)	No RB	—	—	—	D	Y, TCTh, TTh, CryoTh, BrachyTh	Y, 4 IAC 1*	Enucl	A
54	2‐CT (4)	C	Y, BrachyTh	Y	Enucl	No RB	—	—	—	A
55	2‐CT (4)	D	Y, TTh, CryoTh	Y, EBRT	Enucl	D	Y, TTh, CryoTh	Y, EBRT	Enucl	D
56	2‐CT (4)	D	Y, CryoTh	Y	Enucl	B	Y, BrachyTh	Y, 4 IAC 2*	Saved	A
57	2‐CT (4)	A	Y, TTh, CryoTh	N	Saved	D	Y, TTh, CryoTh	N	Saved	A
58	2‐CT (4)	E	N	Y, 4 IAC 2*	Saved	E	N	Y, 4 IAC 2*	Enucl	A
59	2‐CT (4)	D	N	Y, 6 IAC 2*	Saved	D	Y, BrachyTh	N	Saved	A
60	2‐CT (4)	E	N	Y	Enucl	E	N	Y	Enucl	A

Abbreviations: 2‐CT: two drugs (carboplatin/etoposide) – combination chemotherapy; A, Alive; BrachyTh, brachitherapy; CryoTh, criotherapy; D, Dead; EBRT, external beam radiotherapy; enucl, enucleated; IAC, intra‐arterial chemotherapy 1* with Melphalan infusion 2* with Melphalan and Topotecan infusions (Melphalan: 3 mg dose < 12 months of age; 4 mg dose ≥ 12 and < 24 months; 5 mg dose ≥ 36 months; Topotecan 1 mg dose < 12 months; 2 mg dose ≥ 12 months); IVT, intravitreal with melphalan injection (40 microgram dose); N, No; Pt, patient; TCTh, ThermoChemoTherapy; TTh, thermotherapy; Y, Yes.

### 3.2 Chemotherapy Courses and Focal Therapies

All patients received four cycles of carboplatin/etoposide, while 32 out of 60 patients underwent two additional courses of carboplatin in TCTh modality. Chemotherapy was administered every 21 days, except in 10 patients who experienced treatment delays of more than 7 days due to grade 3 neutropenia (median delay: 13 days; range, 8–20 days). However, no patients required dose reductions in subsequent courses. Focal therapy was employed to consolidate non‐calcified tumor regions, at the discretion of the treating ocular oncologist. The choice of focal therapy depended on tumor location: thermotherapy was applied to tumors located posteriorly or anteriorly to the equator of the globe; cryotherapy was used for tumors closer to the *ora serrata*. A detailed overview of treatments is detailed in Table 2.

### 3.3 Outcomes

**FIGURE 1:**
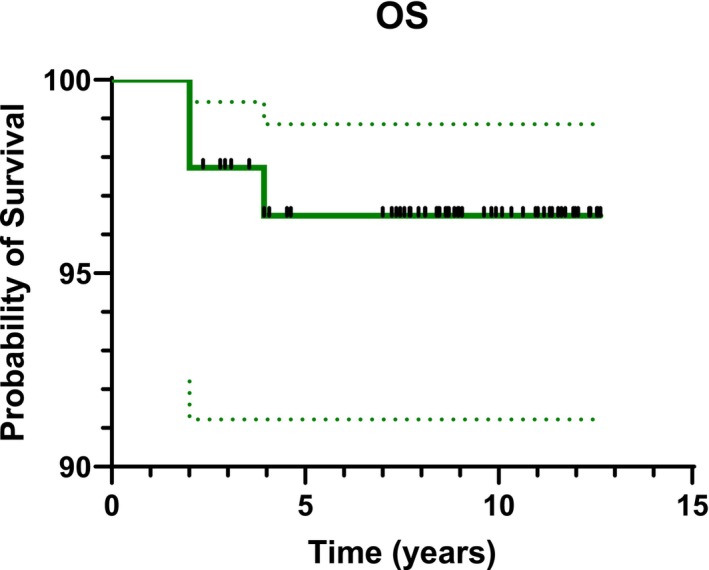
Patients' overall survival (OS).

**FIGURE 2:**
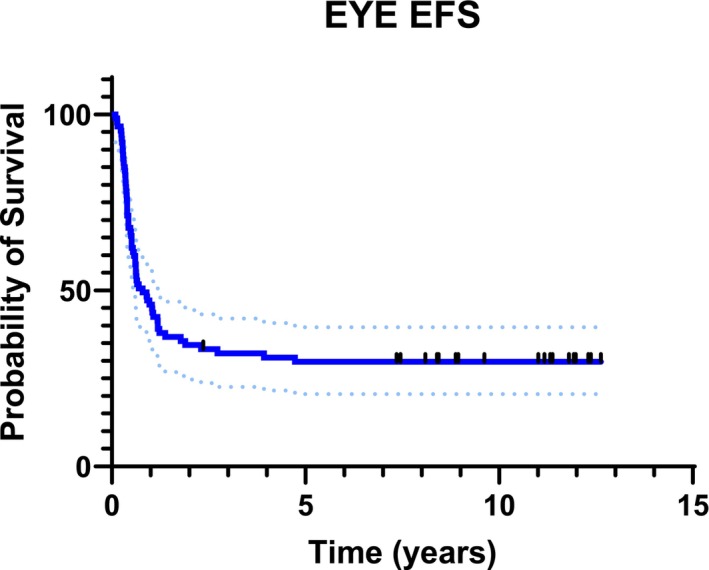
Global eye event free survival (EFS).

With a median follow‐up of 8.71 years ranging from 2 to 12.6 years, the OS for the whole patients' cohort was 96.6% (95% CI: 89.8–98.9) (Figure 1). Eye EFS at 1 to 2 and 5 years was 43.2% (95% CI: 32.7–53.2), 31.1% (95% CI: 24.4–44.0), and 29.5% (95% CI: 20.4–39.2) respectively (Figure 2).

**FIGURE 3:**
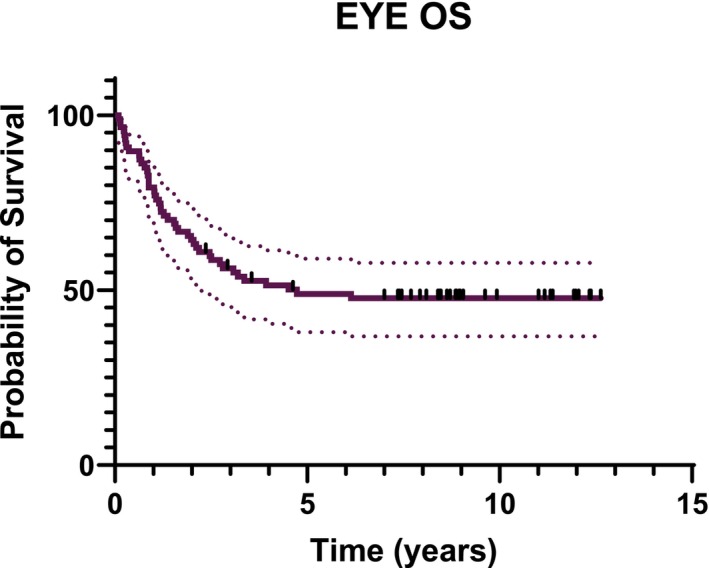
Global eye overall survival (OS).

**FIGURE 4:**
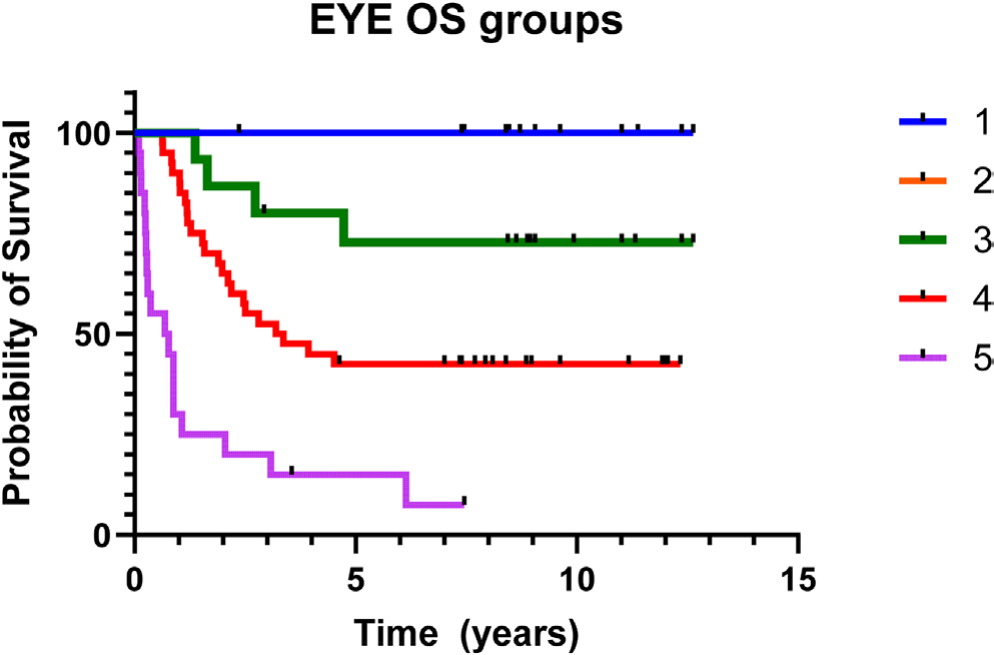
Eye overall survival (OS) stratified by international intraocular retinoblastoma classification‐IIRC (1 = group A; 2 = group B; 3 = group C; 4 = group D; 5 = group E).

Eye OS at 1 to 2 and 5 years was 76.1% (95% CI: 65.8–83.7), 63.3% (95% CI: 52.7–72.7) and 48.9% (95% CI: 38.1–58.8), respectively (Figure 3). The stratification of OS according to the IIRC revealed statistically significant differences. At 1 year, the Eye OS was 100.0% for A, B, and C‐IIRC group, 90.0% (95% CI: 75.5–96.1) for D‐IIRC group, and 28.6% (95% CI: 11.7–48.2) for E‐IIRC group. At 2 years, the Eye OS was 100.0% for A and B, 86.7% (95% CI: 56.4–96.5) for C, 65.0% (95% CI: 48.2–77.6) for D, and 23.8% (95% CI: 8.7–43.1) for E. At 5 years, the Eye OS was 100.0% for A and B, 73.3% (95% CI: 43.6–89.1) for C, 42.5% (95% CI: 27.1–57.0) for D, and 14.3% (3.6%–32.1%) for E (Figure 4). Forty‐six eyes/88 (52%) underwent enucleation for failure of conservative treatment: 21 eyes in 32 patients with unilateral Rb (group C‐IIRC *n* = 3 eyes; group D‐IIRC *n* = 18 eyes) and 25 eyes in 28 patients with bilateral Rb (group C‐IIRC *n* = 1 eye; group D‐IIRC *n* = 5 eyes, group E‐IIRC *n* = 19 eyes). Six out of 28 bilateral patients (21%) underwent bilateral enucleation, and among these, one patient underwent bilateral EBRT before enucleation. No other eye underwent EBRT. Salvage treatments with IAC and IVCT enabled the preservation of 13 eyes that had failed first‐line treatments and that were otherwise destined for enucleation. Among the eyes preserved with second‐line therapies: 6/13 eyes were classified as group D‐IIRC, 2 eyes group E, 4 eyes group C, and 1 eye group B. Twenty patients required additional systemic chemotherapy after enucleation for high‐risk histopathological factors: post‐laminar optic nerve invasion‐N2 (*n* = 2 eyes/2 patients); surgical margin invasion of the optic nerve‐N3 (*n* = 3 eyes/3 patients); massive choroidal invasion‐C2 (*n* = 16 eyes/13 patients), two patients had more than one high‐risk histopathological factor (N2 and C2 *n* = 3 eyes/2 patients). Three patients developed central nervous system (CNS) metastatic disease at 13, 14, and 24 months after primary diagnosis, respectively. Among them, 2 patients out of 3 had CNS dissemination during salvage IAC. Despite intensive treatment with high dose thiotepa/carboplatin/etoposide and cranio‐spinal radiotherapy, all three patients died at 12, 11, and 16 months after relapse, respectively. No secondary, non‐ocular malignancy was reported at our last follow‐up.

### 3.4 Toxicity

Pancytopenia, specifically grades 3 and 4, was the predominant adverse event noted in the patient cohort. The hematological recovery was satisfactory, and no patient necessitated a dose change in the following treatment cycle. One patient presented grade 1 ototoxicity. Other hematological and systemic toxicities are detailed in Table 3. Concerning local ocular toxicity, at the most recent follow‐up, only 6 eyes out of 42 preserved (14%) exhibited problems. Cataract was observed in combination with salt‐and‐pepper retinopathy in one eye of a patient with bilateral Rb. Salt‐and‐pepper retinopathy was observed in one eye of 3 bilateral Rb patients and 2 unilateral Rb patients.

**TABLE 3:** Systemic toxicities (according to CTCAE v4.0).

Adverse events	Number of patients	Number of episodes grade 1–2	Numbers of episodes grade 3	Numbers of episodes grade 4
Anemia	60	50	31	0
Thrombocytopenia	60	30	30	33
Neutropenia	60	60	20	33
Febrile neutropenia	22	0	35	0
Diarrhea	12	10	2	0
Mucositis	40	40	20	0
Vomiting	60	80	0	0
Dehydration	0	0	0	0
Allergic reactions	0	0	0	0

### 3.5 Visual Outcome

At a median age of 10.1 years (range 4.1–14.9 years), 42 preserved eyes (22 from patients with bilateral Rb and 11 from unilateral Rb) who completed therapy had documented vision: 28/42 eyes had measurable vision (range: 20/20–20/400, mean 20/40, according to Snellen VA chart). Notably, 13/28 eyes achieved a VA of 20/40 or better. In contrast, 14/42 eyes had such severely impaired VA that it could not be quantified. Among these, 9/14 eyes belonged to unilateral Rb patients and 5 eyes to bilateral Rb, the other eye having a VA of at least 20/30. Among 22 patients with bilateral disease and at least one eye preserved, 12 patients (age at diagnosis ranging from 3 to 23 months‐median 5.5 months) had a documented binocular vision (vision with the better‐seeing eye) of 20/30 or better. In 10/12 patients, at least one eye had a less advanced disease at diagnosis and was classified as group A/B according to IIRC. Patients' VA details are reported in Table 4.

**TABLE 4:** Visual acuity according to Snellen Chart.

Pt	IIRC group right eye	Final VA	IIRC group left eye	Final VA
1	C	20/200	A	20/20
2	A	20/200	E	Enucleated
3	E	Enucleated	C	Enucleated
4	No Rb	—	D	Enucleated
5	D	Enucleated	No Rb	—
6	D	Enucleated	No Rb	—
7	A	20/20	C	20/400
8	D	20/200	No Rb	—
9	D	20/200	E	Enucleated
10	No Rb	—	D	Count fingers
11	C	Enucleated	No Rb	—
12	D	Enucleated	No Rb	—
13	E	Enucleated	B	20/20
14	No Rb	—	C	Count fingers
15	No Rb	—	D	Count fingers
16	No Rb	—	D	Enucleated
17	D	Enucleated	No Rb	—
18	D	Enucleated	No Rb	—
19	D	Enucleated	No Rb	—
20	No Rb	—	D	Enucleated
21	C	20/200	E	Enucleated
22	No Rb	—	D	Enucleated
23	D	Enucleated	No Rb	—
24	E	Enucleated	E	Enucleated
25	A	20/20	D	Light perception
26	C	Count fingers	B	20/20
27	E	Enucleated	D	20/30
28	C	Hand motion	No Rb	—
29	C	Count fingers	No Rb	—
30	E	Enucleated	D	20/100
31	C	Enucleated	No Rb	—
32	E	Enucleated	E	Enucleated
33	C	20/100	E	Enucleated
34	E	Enucleated	C	20/80
35	D	Enucleated	E	Enucleated
36	D	20/250	E	Enucleated
37	No Rb	—	D	Enucleated
38	No Rb	—	D	Hand motion
39	D	Enucleated	No Rb	—
40	E	Count fingers	B	20/30
41	D	Light perception	A	20/20
42	C	Light perception	No Rb	—
43	E	Enucleated	D	20/30
44	D	Light perception	No Rb	—
45	No Rb	—	D	Light perception
46	No Rb	—	D	Enucleated
47	A	20/20	D	Enucleated
48	D	Enucleated	No Rb	—
49	No Rb	—	D	20/40
50	C	20/200	A	20/25
51	No Rb	—	D	Enucleated
52	No Rb	—	D	Enucleated
53	No Rb	—	D	Enucleated
54	C	Enucleated	No Rb	—
55	D	Enucleated	D	Enucleated
56	D	Enucleated	B	20/100
57	A	20/20	D	Hand motion
58	E	20/200	E	Enucleated
59	D	20/200	D	20/200
60	E	Enucleated	E	Enucleated

Abbreviations: Pt, patient; Rb, retinoblastoma; VA, visual acuity.

## 4 Discussion

This study presents the outcomes of 60 patients (88 eyes) affected by intraocular Rb and enrolled in the AIEOP RTB 012 protocol, high risk group, between 2012 and 2017. Ocular EFS rates were 43.2% at 1 year, 31.1% at 2 years, and 29.5% at 5 years (Figure 2). Notably, 31% of preserved eyes (13 out of 42) were successfully saved through second‐line therapies, including IAC and IVTC (Table 2). The ocular OS rates were, therefore, 76.1% at 1 year, 63.3% at 2 years, and 48.9% at 5 years (Figure 3). When stratified by IIRC, ocular OS revealed statistically significant differences among the groups. At 2 and 5 years, ocular OS rates were 100% for groups A and B, while they decreased to 86.7% and 73.3% for group C, 65% and 42.5% for group D, and 23.8% and 14.3% for group E, respectively (Figure 4). These findings emphasize that early diagnosis and treatment are crucial, as more advanced stages (groups D and E) are associated with significantly lower ocular survival rates. Our results align with those of previous studies, such as the 2006 study by Shields et al., which evaluated the efficacy of chemoreduction (CRD) using the CEV regimen (vincristine, etoposide, and carboplatin) for treating retinoblastoma. This study also demonstrated variability in ocular preservation success depending on the ICRB group. In their findings, Group A achieved a 100% success rate with CRD, while Groups B and C showed success rates of 93% and 90%, respectively. However, for Group D, the success rate dropped to 47%, often necessitating additional treatments like external beam radiotherapy (EBRT) or even enucleation [11]. Our study corroborates these findings, showing that the carboplatin/etoposide regimen is highly effective for early‐stage retinoblastoma (Groups A–C) with minimal systemic toxicity, although different treatments are required for advanced cases. A recent meta‐analysis by Daniels and colleagues, which synthesized data from 27 studies involving 1483 eyes, further supports these findings. Their report on globe salvage rates with systemic carboplatin/etoposide regimens plus focal treatments showed a similar decline in ocular survival as the disease progresses. Specifically, Group A had a 93% salvage rate, Group B had 83%, Group C had 73%, Group D had 40%, and Group E had just 19%. This pattern reinforces our observation that advanced disease stages result in lower ocular salvage rates [12]. However, even if this chemotherapy regimen has proved to be effective for early‐stage retinoblastoma, the lower success rates in more advanced stages emphasize the need for additional treatment strategies. In recent years, IAC demonstrated to be more efficacious than systemic chemotherapy, both as first and second‐line treatment, in eyes with advanced intraocular disease. A 15‐year experience with IAC reported that 61% of treatment‐naïve group E‐IIRC eyes were salvaged, while 75% of the group E‐IIRC eyes that had failed prior treatment were successfully preserved [13]. The same trend was observed for group D‐IIRC eyes, as highlighted in a meta‐analysis of 613 studies [14]. Moreover, the safe IVTC technique, introduced by Munier in 2012 [15] allows for the treatment of advanced intraocular disease with refractory/recurring vitreous seeding that would otherwise have been enucleated. In agreement with these data, in our patients' cohort, IAC and IVTC allowed for the recovery of 13 eyes, over half of which presented with very advanced disease at diagnosis: 6/13 in group D; 2/13 in group E. While these treatments have demonstrated high efficacy in controlling the tumor, they have raised concerns about potential visual impairments. Studies have shown that, although IAC and IVTC are more effective in tumor control, they can lead to significant ocular toxicities that may not be associated with systemic chemotherapy. A recent retrospective study, conducted at Vanderbilt University, compared IAC and intravenous chemoreduction (IVC) for treating retinoblastoma, focusing on ocular complications and visual outcomes. While IAC demonstrated a significantly higher globe salvage rate of 100% over 3 years as compared to 58% for IVC (*p* = 0.021), it was associated with a higher incidence of certain ocular complications, including intra‐tumoral or subretinal hemorrhage and long‐lasting chorioretinopathy (*p* = 0.02) [16]. Another study conducted by Tsimpida and colleagues retrospectively examined the ocular complications and visual outcomes associated with intra‐arterial melphalan therapy in 12 eyes from 12 patients with refractory retinoblastoma. While IAC demonstrated a tumor control rate of 75%, the study found that 42% of the treated eyes experienced severe visual impairment, primarily due to complications, such as retinal detachment (20%) and choroidal ischemia affecting the foveola (80%). Additionally, 50% of the eyes showed a decline in function, as indicated by post‐treatment electroretinograms, suggesting a significant risk of widespread retinal toxicity [17]. In line with the data mentioned above, in our patients' cohort, most ocular toxicities were observed among eyes rescued with second‐line IAC and IVTC: in fact, 5 out of 13 preserved eyes (38%) presented salt‐and‐pepper retinopathy (4 eyes), both cataract and salt‐and‐pepper retinopathy (1 eye). In another study by Riazi‐Esfahani and collaborators, they examined the effects of chemotherapy on the retinal microvascular structure in children with Rb. The study utilizes advanced imaging techniques, specifically Optical Coherence Tomography Angiography (OCTA) and Enhanced Depth Imaging Optical Coherence Tomography (EDI‐OCT), to investigate retinal and choroidal changes in 28 eyes, after IAC (12 eyes) and IVC (19 eyes). Patients receiving IAC had poorer visual acuity (mean 1.03 logMAR) compared to those treated with IVC (mean 0.46 logMAR), indicating a correlation between treatment type and visual outcomes. Central macular thickness and subfoveal choroidal thickness were lower in the IAC group compared to their contralateral eyes and normal controls (*p* < 0.05), suggesting potential choroidal atrophy due to the chemotherapeutics used in IAC [18]. Regarding our patients' cohort visual outcome, at a median age of 10.1 years at the time of evaluation, 12 out of 28 patients with bilateral disease (42.8%) achieved a binocular VA of at least 20/30 according to the Snellen scale, allowing them to perform most daily activities without significant difficulty. The majority of these patients had an unbalanced intraocular staging of disease between the two eyes; in fact, 10 out of 12 had one eye with low disease burden (group A/B‐IIRC), which was preserved with systemic chemotherapy in association with focal treatments, without resorting to IAC or second‐line IVTC. In comparison, a 2020 study from Institut Curie reported that 37.5% of bilateral Rb patients treated with intravenous chemoreduction and focal treatments achieved a binocular visual acuity better than 20/40, a result similar to ours [19]. A study by Lumbroso‐Le Rouic et al., published in 2021, reported less favorable VA outcomes in patients treated with primary IAC for unilateral Rb (groups B/C/D‐IIRC): only 2 out of 20 evaluable patients achieved a VA greater than 20/30 and 6 additional patients had a measurable VA of less than 20/100 [20]. However, the impact of chemotherapy on final visual outcomes remains a significant concern, particularly for patients with bilateral disease, where both ocular preservation and visual function are crucial endpoints. This highlights the need for further research into the balance between tumor control and visual outcomes, as well as the potential role of IAC and IVTC as rescue therapies for advanced or recurrent disease. Moreover, while the conservative approach to treatment was generally well tolerated in our cohort, the risk of extraocular dissemination remains a critical concern. In our study, 3 patients developed central nervous system (CNS) progression during second‐line IAC. The prognosis for CNS metastatic retinoblastoma is poor, with a five‐year overall survival rate below 15%. This reinforces the importance of careful monitoring for signs of CNS spread, particularly in cases where the disease is refractory to initial treatments [21]. A retrospective study by Munier et al. analyzed data from six Rb centers worldwide and reported three CNS metastases among 1139 patients treated with IAC over 10 years. While the incidence is reassuringly low, careful and rigorous monitoring is necessary due to the severe consequences of extraocular progression [22]. Finally, the identification of high‐risk prognostic factors, such as MYCN amplification and increased Trefoil Factor‐1 (TFF1) expression, is crucial for improving risk stratification and treatment decisions [23, 24, 25, 26]. While these factors are typically assessed after enucleation, recent advances in radiogenomics and liquid biopsy may offer non‐invasive alternatives for early molecular characterization of the tumor, potentially enhancing personalized treatment strategies [27, 28, 29]. Moreover, neo‐adjuvant chemotherapy does not eliminate high‐risk histoprognostic factors in enucleated eyes. In our study, among 40 patients who underwent enucleation, 50% of them received additional adjuvant chemotherapy due to high‐risk histopathological findings [30]. In our cohort, only one patient with bilateral Rb required EBRT for both eyes. This finding is particularly relevant in patients with a germline Rb1 mutation who have a markedly elevated risk of developing radiation‐induced secondary cancers [31]. No secondary non‐ocular malignancies were reported in our patients at the last follow‐up. In line with the results reported by Shields et al. [11], in our patients cohort, systemic CRD was well tolerated, with no severe adverse events. Regarding carboplatin‐related ototoxicity, in our study, no patient experienced hearing toxicity greater than grade 1, a relevant finding in these children already facing sensory organ challenges. In this regard, a report from St. Jude showed a higher incidence of hearing loss after carboplatin (20% of patients, most grade 3 or 4 and bilateral). This finding can be attributed to several factors: a higher proportion of patients under 6 months at the start of treatment (who are more susceptible to developing hearing loss compared to older patients); carboplatin dosing based on body surface area (mg/m^2^), which may have resulted in increased drug exposure for younger patients; a greater number of carboplatin cycles (with a median of 8 cycles) compared to our protocol that typically includes 6 cycles at most [32].

## 5 Conclusions

In conclusion, our data suggest that the conservative treatment strategy employed for children with intraocular retinoblastoma is well tolerated and leads to satisfactory visual outcomes at long‐term follow‐up. However, caution is necessary due to the potential risk of dissemination to the CNS during prolonged conservative treatment. Second‐line IAC and IVTC have proven effective in enhancing treatment success, particularly in advanced disease, and will be integrated into frontline therapy in future prospective protocols for patients with unilateral disease. While these treatments are highly effective in controlling the tumor, their impact on final visual outcomes, particularly in bilateral cases, remains a challenge and warrants further investigation. Moreover, extended follow‐up is essential for assessing the long‐term consequences of treatment, including the risk of secondary non‐ocular malignancies.

## Author Contributions

**Ida Russo:** conceptualization, investigation, writing – original draft, methodology, validation, visualization, data curation, resources, formal analysis, writing – review and editing. **Valentina Di Ruscio:** investigation, data curation, resources, writing – original draft. **Maria Antonietta De Ioris:** conceptualization, investigation, methodology, validation, data curation, supervision, resources, writing – review and editing. **Giada Del Baldo:** investigation, methodology, writing – original draft, formal analysis, data curation, supervision. **Maria Debora De Pasquale:** supervision, data curation. **Paola Valente:** conceptualization, investigation, writing – original draft, methodology, validation, formal analysis, data curation, supervision, resources, writing – review and editing. **Enrico Opocher:** data curation, supervision, investigation. **Raffaele Parrozzani:** data curation, supervision. **Angela Di Giannatale:** supervision. **Maria Giuseppina Cefalo:** data curation, investigation. **Annalisa Serra:** data curation. **Giuseppe Maria Milano:** supervision. **Daniela Longo:** data curation. **Alessia Carboni:** data curation. **Rita De Vito:** data curation, investigation. **Gianluigi Natali:** investigation, data curation, supervision. **Gemma D'Elia:** investigation. **Gianni Bisogno:** investigation, supervision, data curation. **Angela Mastronuzzi:** conceptualization, investigation, methodology, validation, data curation, supervision, writing – review and editing. **Raffaele Cozza:** conceptualization, investigation, methodology, validation, data curation, supervision. **Rita Alaggio:** supervision. **Antonino Romanzo:** data curation, conceptualization, investigation, methodology, validation. **Luca Buzzonetti:** investigation, supervision, data curation. **Franco Locatelli:** conceptualization, investigation, methodology, validation, data curation, supervision, writing – review and editing.

## Ethics Statement

This study was conducted in accordance with the principles of the Declaration of Helsinki. Ethics approval was obtained from the “Bambino Gesù Ethics Committee”, protocol number 2011‐006109‐85.

## Consent

Informed consent was obtained from all individual participants included in the study. For participants under 18 years of age, written informed consent was obtained from a parent or legal guardian.

## Conflicts of Interest

The authors declare no conflicts of interest.

## Data Availability

The data that support the findings of this study are available from the corresponding author upon reasonable request. All data generated or analyzed during this study are included in this published article and its Supporting Information.
